# Modified Corona Score can easily identify Covid-19 patients with gastrointestinal symptoms: An Italian proposal 

**Published:** 2020

**Authors:** Roberto Assandri, Alessandro Montanelli

**Affiliations:** 1 *Clinical Investigation Laboratory, Clinical Investigation Laboratory, ASST-Crema, Cremona, Italy*; 2 *Clinical Investigation Laboratory, Ospedale Bolognini Seriate, Bergamo, ASST-Seriate, Bergamo Italy *

**Keywords:** Modified Corona Score, Covid-19, RT-PCR, Gastrointestinal onset, Laboratory parameters

## Abstract

**Aim:**

We propose the Modified Corona Score (MCorona score), an alternative approach to identifying new likely Covid-19 patients without positive chest images, but with gastrointestinal onset.

**Background:**

In April, 2020, a total of 104,291 laboratory-confirmed cases had been documented in Italy; Lombardy, the Northern Italian Region, recorded over 60,000 Covid-19 cases.

**Method:**

The MCorona score is built by several laboratory parameters linked between age and gender, ranging from 0 to 10.

**Results:**

Using the preliminary score cut-off of 4, we successfully identified likely Covid-19 patients with gastrointestinal onset. However, more caution is needed, and a larger sample size is required to verify the accuracy and specificity of the score.

**Conclusion:**

We propose the complete validation of the MCorona score, an instrument able to diagnose likely Covid-19 patients with symptoms other than respiratory distress.

## Definition

 On behalf of all authors, the corresponding author states that there is no conflict of interest.

After the December 2019 outbreak in China, the novel Coronavirus infection, designated SARS-CoV-2 (Covid-19), spread very quickly worldwide. By April, 2020, a total of 104,291 laboratory-confirmed cases had been documented in Italy.

Large-scale testing finds and isolates infections quickly, limiting the virus’s spread and protecting vulnerable populations. To properly survey populations, millions of Covid-19 test kits will need to be processed. Organizations around the world are trying to ramp up their capacity as quickly as possible. Local, national, and international media continuously attack regional and national institutions, complaining about the inability of the Lombardy Region, the most solid and advanced Italian region in terms of public health, for not having the possibility to process a capillary population-based swab screening test. 

Covid-19 causes a clinical syndrome encompassing a wide range of clinical features, from an asymptomatic or oligosymptomatic course to acute respiratory distress and death (1, 2).

In a very recent publication in Gut, Lin et al. showed that around 11% of patients infected with Covid-19 presented with gastrointestinal (GI) symptoms (1), i.e. diarrhea, nausea, vomiting, and/or abdominal pain, or described having them at disease onset or even before respiratory symptoms. Following this evidence, our work published in Gut clearly remarked the importance of including GI symptoms in the spectrum of Covid-19 features (3). 

In a very recent work, we preliminarily observed that several laboratory tests have been shown as characteristically altered in Covid-19. In our paper, baseline biochemical parameters were clearly linked to clinical features. Our hematological exams at admission showed low white blood cell counts (WBC count) and low neutrophil counts in over 80% of cases and lymphocyte counts below 1x109/L at least half of the patients. Also C-reactive protein (CRP) serum levels were elevated in most patients, particularly among those with the worst clinical picture and outcome (Benelli G et al., SARS-COV-2 comorbidity network and outcome in hospitalized patients in Crema, Italy. https://doiorg/101101/2020041420053090 2020). 

In our opinion paper published in the Archive of Medical Research, we proposed a few laboratory tests as rapid and sensitive alternatives for identifying likely Covid-19 cases (4). 

The Corona Score model was recently proposed as an easy-to-use algorithm for identifying Covid-19 patients with respiratory symptoms (5). This approach uses a chest images-relative score (1 to 4) and several laboratory parameters to classify emergency room patients. This algorithm is recommended for use only with patients with respiratory symptoms and fails in patients with negative chest imaging, as occurs in some Covid-19 patients with GI onset (5).

In the light of these observations, we decided to choose an “alternative” approach to identifying likely Covid-19 patients with GI onset. 

We have created and propose a new scoring system, called the Modified Corona Score, (MCorona score), using age, gender, and laboratory parameters, as shown in table 1, without including chest images as part of the score that now ranges between 0 and 10.

We retrospectively analyzed 400 confirmed Covid-19 patients (with positive real-time polymerase chain reaction) admitted to the General Hospital of Crema between February 21 and March 13, 2020. Among them, 42 (10.5%) reported GI symptoms including nausea vomiting, diarrhea, and abdominal pain. Absence of cough was reported in 83% of patients with GI symptoms and 16.6% (7 patients) with negative chest imaging.

We applied our score to a patients cohort and calculated the score range and mean value. The MCorona score in our patients cohort ranged between 6 and 10, with the mean score being 7. The same evidence was reported in 42 patients with GI symptoms (6-10, mean = 7). Using preliminary score cut-offs of 4, we applied this evidence to patients without respiratory onset. All six patients presented a mean range of 7, the same as other patients. The preliminary score accuracy appeared satisfactory, increasing the detection rate of patients with GI onset and negative chest images (42/42, 100%).

Our study offers a new and rapid tool for identifying patients with gastrointestinal onset of Covid-19. This scoring method could be used to accelerate determination of isolation needs and optimize the predictive outcome of CT scans. However, more caution and a larger sample size are needed in order to verify the accuracy and specificity of the score.

In conclusion, we propose the completely validated MCorona score method for assessing Covid-19 patients with GI features. The preliminary data regarding this scoring system is very encouraging, but further studies are needed. MCorona score could open a new road to successfully identifying all likely Covid-19 patients with symptoms other than respiratory distress. 

**Table 1:** Modified Corona Score

Age (years)	≤75	76-79	80				
Point	0	1	2				
Sex (M/F)	Female	Male					
Point	0	1					
CRP (mg/dL)	0 to 0.09	1 to 4	1.5 to 3.8	3.9 to 6.9	7 to 19	19.4 to 30.3	30.4 to 50
Point	0	1	2	3	2	1	0
LDH (U/L)	≤257	258-265	266-397	≥398			
Point	0	1	2	3			
ALC (10^9^/L)	≤1.2	1.3					
Point	1	0					
ANC (10^9^/L)	≤5.1	5.2-7.9	8.0-9.0	9.1-10.3	≥10.4		
Point	0.00	-1.00	-2	-3	-4		

CRP, C-reactive protein; LDH, lactate dehydrogenase; ALC, absolute lymphocyte count; ANC, absolute neutrophil count; CXR, chest X-ray

## Conflict of interests

The authors declare that they have no conflict of interest.
